# Important Role of Autophagy in Endothelial Cell Response to Ionizing Radiation

**DOI:** 10.1371/journal.pone.0102408

**Published:** 2014-07-10

**Authors:** Dimitra Kalamida, Ilias V. Karagounis, Alexandra Giatromanolaki, Michael I. Koukourakis

**Affiliations:** 1 Department of Radiotherapy/Oncology, Democritus University of Thrace, and University General Hospital of Alexandroupolis, Alexandroupolis, Greece; 2 Department of Pathology, Democritus University of Thrace, and University General Hospital of Alexandroupolis, Alexandroupolis, Greece; School of Medicine, University of Belgrade, Serbia

## Abstract

**Objectives:**

Vasculature damage is an important contributor to the side-effects of radiotherapy. The aim of this study is to provide insights into the radiobiology of the autophagic response of endothelial cells.

**Methods and Materials:**

Human umbilical vascular endothelial cells (HUVEC) were exposed to 2 Gy of ionizing radiation (IR) and studied using confocal microscopy and western blot analysis, at 4 and 8 days post-irradiation. The role of autophagy flux in HUVEC radio-sensitivity was also examined.

**Results:**

IR-induced accumulation of LC3A+, LC3B+ and p62 cytoplasmic vacuoles, while in double immunostaining with lysosomal markers (LAMP2a and CathepsinD) repression of the autophagolysosomal flux was evident. Autophagy-related proteins (ATF4, HIF1α., HIF2α, Beclin1) were, however, induced excluding an eventual repressive effect of radiation on autophagy initiating protein expression. Exposure of HUVEC to SMER28, an mTOR-independent inducer of autophagy, enhanced proLC3 and LC3A, B-I protein expression and accelerated the autophagic flux. Pre-treatment of HUVEC with SMER28 protected against the blockage of autophagic flux induced by IR and conferred radio-resistance. Suppression of LC3A/LC3B proteins with siRNAs resulted in radio-sensitization.

**Conclusions:**

The current data provide a rationale for the development of novel radioprotection policies targeting the autophagic pathway.

## Introduction

Endothelial cell damage is a major effect of radiotherapy. Increased permeability leads to oedema and inflammation of organs which contributes to acute side effects. Vascular and lymphatic damage is also a main component of the unpredictable and sometimes life-threatening late side effects, appearing even years after radiotherapy [1], [2], [3]. Arm oedema or haemorrhage from extensive formation of telangiectasia of the bladder or rectal mucosa are the extremely problematic for patients and clinicians. Cytoprotection policies alleviating the radiation-induced vascular damage may improve the therapeutic index of radiotherapy [4].

The vast majority of research efforts focus on agents protecting from DNA damage. Nevertheless, biological processes residing in the cytoplasm may also play an important role. Macro-autophagy (autophagy), is an important biological process responsible for the turnover and recycling of long-lived proteins and dysfunctional organelles. This could have an important role in the elimination of damage by intracellular structures exposed to radiation [5], [6]. Autophagic vacuoles fuse with lysosomes, resulting in digestion of the content, and subsequently release molecules to be re-used as a metabolic fuel. Abnormal function of autophagy may interfere with the protein and organelle quality control in irradiated cells, which may lead to cell death or degeneration followed by cellular and tissue dysfunction [7].

Here, we investigated the effect of a clinically relevant dose of IR on the autophagic machinery of human endothelial cells using confocal immnofluorescent microscopy and western blot analysis for multiple autophagy-related proteins. The effect of silencing the LC3A and B genes, and that of autophagy induction using the SMER28 (Small-Molecule Enhancer of Rapamycin-28) mTOR independent agent, on endothelial cell sensitivity is investigated [8].

## Materials and Methods

### Cell Cultures

HUVEC (human umbilical vein endothelial cells) were purchased from CLS (Cell Line Service). Cells were maintained and cultured in EBM-2 Basal Medium (Endothelial basal medium-2, Lonza) with EGM-2 SingleQuots of Growth Factors (Lonza) at standard conditions: 37°C, 5% CO_2_ in humidified atmosphere. Prior to culture, flasks were coated with a 0.2% gelatin solution (ScienCell Research Laboratories) to facilitate adherence.

### SMER28

SMER28 was purchased from ENZO Life Sciences. Stock solution was prepared at its maximum solubility at 8 mg/ml in DMSO according to the manufacturer. The concentrations of 25 and 50 µΜ have been selected for our experimental procedure based on viability and titration tests.

### Cell survival and regrowth experiments

Cells were placed in 96-well plates at a concentration of 250 cells/well. Irradiation of the plates was performed using a 6 MV beam of a Linear Accelerator (PRECISE; ELEKTA) endowed with a MultiLeaf collimator. For multidose irradiation of the well columns within the same 96-well plate, a previously validated and reported technique was used [9]. Cell proliferation and survival experiments were performed using the AlamarBlue Cell Viability Reagent (DAL1100, Invitrogen) assay, as previously validated by our group [10].

### Immunoblotting

For the autophagic characterization under radiation exposure, HUVEC cells were cultured at standard conditions: 37°C, 5% CO2 in humidified atmosphere. The conditions tested include the irradiation of cells at 2 Gy and the collection of protein lysates after 4 and 8 days post-irradiation. For the study of the SMER28 effect, with or without the irradiation influence, the conditions tested include a) treatment of cells with 50 µM SMER28 for 3days and b) treatment with SMER28 (same conditions as before) as well as irradiation at 2 Gy and collection of protein lysates after 4 and 8 days post-irradiation. Cells were irradiated utilizing a Co60 unit.

Cells were washed with PBS twice and lysed in a sucrose-based lysis buffer (0.25 M sucrose, 25 mM Tris-HCl, pH 7.4) containing protease inhibitors (complete mini protease inhibitor cocktail, Roche Diagnostics GmbH) and phosphatase inhibitors (phosphatase inhibitor cocktail, Cell Signaling Technology). A differential centrifugation of the whole-cell lysates led to supernatant (cytoplasmic-water soluble proteins) and pellet (membrane proteins) fractions. Protein quantification was performed according to the Pierce BCA Protein Assay Kit (#23225, Thermo Scientific). A total of 40 µg (for LC3A, LC3B, P62, ATF4, cathepsin D, Lamp2a) and 80 µg (for Beclin1, HIF1a) of each protein lysates were resolved by discontinuous SDS gels using 15% (LC3A, LC3B), 12% (p62, cathepsin D, ATF4, Beclin1) and 8% (LAMP2a, HIF1a) separation and 5% stacking gels, and they were transferred to the appropriate PVDF membrane. All different experimental condition lysates were loaded on the same gel and transferred on the same membrane. Following blocking with TBS (pH 7.6) containing 0.1% (v/v) Tween 20 and 5% (w/v) non-fat dried milk for 1 h at room temperature, membranes were hybridized at 4°C overnight with primary anti-MAP1LC3A (1∶1000, ab62720, Abcam), anti-MAP1LC3B (1∶1000, 0231-100/LC3-5F10, Nanotools), anti-SQSTM1/p62 (1∶1000, ab64134, Abcam), anti-ATF-4 (phospho S245) (1∶500, ab28840, Abcam), anti-Cathepsin D [CTD-19] (1/500, ab6313, abcam), anti-Beclin1 (1/500, ab62557, Abcam), anti-LAMP2a (1/500, ab18528, Abcam), anti-HIF1α (1/500, NB100-105, Novus Biologicals) and anti-HIF2α/EPAS1 mouse monoclonal (1∶2; gift from Professor K.C. Gatter, Oxford UK) antibodies. Then, the membranes were hybridized with the appropriate secondary antibodies (1∶5000, Goat Anti-Rabbit IgG (H+L)-HRP Conjugate, #170-6515, Biorad and 1∶5000 bovine anti-mouse IgG-HRP, P0447, DAKO) and developed in Amersham ECL Western blotting detection reagents and analysis system (RPN2209, GE Healthcare). Each of these blots was then stripped, and the membranes were reprobed with a primary anti-beta actin antibody (1∶5000, ab75186, Abcam). The images of the blots were captured utilizing Chemidoc MP imaging system (Biorad).

### siRNA

LC3A siRNAs were pooled as (5′-GCGAGUUGGUCAAGAUCAUTT-3′), (5′- GCUUCCUCUAUAUGGUCUATT-3′), (5′-CCUGCUGUGUGG UUCAUCUTT- 3′), (5′-GCUGUAAGGAGGUAC AGCATT-3′), and LC3B siRNA were respectively pooled as (5′-GCCCUCUACUGAUUGUUAATT-3′), (5′-CUCCCUAAGAGGAUCUU-UATT-3′), (5′- GCCUGUGUUGUUACGGAAATT - 3′). These were custom synthesized from Shanghai GenePharma Co., Ltd (China). These were used at 50 nM to transfect HUVECs using HiPerfect (QIAGEN) for 24 h, while the silencing efficiency of siRNAs was confirmed both by confocal microscopy and western blot after 24 h.

### Confocal immunofluorescence

For immunofluorescence staining, cells were grown on No. 1.5 glass coverslips and were fixed in 3.7% formaldehyde/PBS pH 7.4 for 20 min at 37°C, and then they were permeabilized in PBS/0.1% v/v Triton X-100 pH 7.4 for 5 min at room temperature. Cells were blocked in PBS/5% w/v BSA pH 7.4 and stained with various combinations of the following for 1 h at room temperature: anti-MAP1LC3A rabbit polyclonal (1∶500, ab62720, Abcam), anti-LC3B mouse monoclonal antibody (1∶100, 0231-100/LC3-5F10 Nanotools), anti-p62 mouse monoclonal (1∶100, ab56416, Abcam), anti-LAMP2A rabbit polyclonal (1∶500, ab18528, Abcam), anti-CathepsinD mouse monoclonal (1∶200, ab6313, Abcam), anti-Beclin1 rabbit polyclonal (1∶100, ab62557, Abcam), anti-HIF2α/EPAS1 mouse monoclonal (1∶2; gift from Professor K.C. Gatter, Oxford UK), anti-HIF1α mouse monoclonal (1∶20; gift from Professor K.C. Gatter, Oxford UK), anti-ATF4 rabbit polyclonal (1∶100, ab28830, Abcam) and anti- gamma H2A.X rabbit polyclonal (1∶500, ab11174, Abcam). Cells were washed in PBS pH 7.4, incubated with appropriate CF 488 and 564 secondary antibodies (1∶500; Biotium) at room temperature, and the DNA was counterstained with Hoechst 33342 (1 µg/ml; Sigma-Aldrich). After final washes the coverslips were mounted in a homemade Mowiol mounting medium. Imaging was performed on a customized Andor Revolution Spinning Disk Confocal System built around a stand (IX81; Olympus) with a 100×-1.4 NA lens and a digital camera (Andor Ixon+885) (CIBIT Facility, MBG-DUTH). Image acquisition was performed in Andor IQ 2 software. Optical sections were recorded every 0.3 µm. All confocal microscopy images presented in this work are two-dimensional (2D) maximum intensity projections of z-stack images (ImageJ 1.47v National Institute of Health, USA). Graph presentation is performed using the GraphPad Prism 5.01 statistical package (GraphPad Software Inc., USA).

### Image analysis

Image intensity analysis for the obtained data sets was performed using ImageJ 1.47v (National Institute of Health, USA) software. Image processing macros were developed in order to quantify the levels of the examined protein in the area of interest. In particular, for gamma H2A.X nuclear foci, macros were developed to count the number of nuclear foci and the nuclear area in each cell. Statistical analysis was performed by the 1-way ANOVA test (n≥20 cells for each group, ****p<0.0001). The 2D average projection of z-stack images were quantified using a standard sized square area where integrated intensity values had been measured. Statistical analysis by the 2-way ANOVA test (n≥20 cells for each group, ****p<0.0001) and graph presentation was performed using the GraphPad Prism Version 5.01a statistical package (GraphPad Software Inc., USA).

## Results

### Effect of IR on LC3A and LC3B proteins

**Figure 1:**
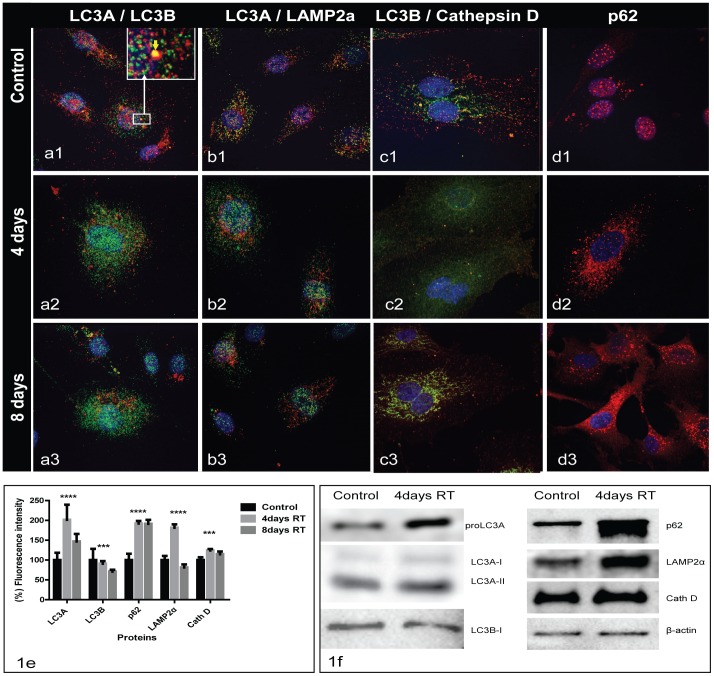
HUVEC confocal immunofluorescent microscopy images LC3A(green)/LC3B(red) (Fig. 1a), LC3A(green)/LAMP2a(red) (Fig. 1b), LC3B(red)/CathepsinD(green) (Fig. 1c) and p62(red) (Fig. 1d). Co-localization is presented as yellow spots. Figure 1a: autophagosomes stained for LC3A (perinuclear localization) and LC3B (cytoplasmic localization). Irradiation-induced accumulation of both LC3A and LC3B autophagosomes at 4 and 8 post-irradiation days (1a2,1a3). Figures 1b1 and 1c1 show an evident degree of co-localization of LC3A with LAMP2a and of LC3B with CathepsinD. At 4 and 8 post-irradiation days there was a lack of co-localization, which suggests autophagy flux suppression (1b2, 1b3, 1c2, 1c3). Figure 1d shows poor cytoplasmic and intense nuclear presence of p62 in control cells (1d1); intense accumulation of p62 in the cytoplasm was evident 4 and 8 days after irradiation (1d2,3). Graphic presentation of fluorescent intensity of the above immunostaining is shown in Figure 1e. Figure 1f shows the western blot analysis in the pellet fraction of HUVEC.

Confocal microscopy at 4 and 8 days following 2 Gy irradiation revealed distinct LC3A and LC3B autophagosomes with lack of co-localization in double staining (Pearson’s coefficient R = 0.16) (**Fig. 1a** **1**). Sporadic LC3B large autophagosomes containing smaller LC3A ones were noted (**Fig. 1a** **1**). LC3A and LC3B autophagosomes had a different subcellular localization; the LC3A showed a nuclear and perinuclear pattern, while LC3B was distributed throughout the cytoplasm (**Fig. 1a** **1**).

At 4 and 8 days following irradiation, there was a dense accumulation of LC3A-positive autophagosomes that was distributed throughout the cytoplasm and strongly within the nuclei (**Figures 1a** **2 and 1a3**). LC3B autophagosome distribution was rather unaffected. These were confirmed with image densitometry (**Figure 1e**).

In western blot analysis, IR induced reduction of the proLC3A form in the supernatant fraction, but was clearly increased in the pellet fraction (LC3A-II form) (**Figure 1f**). There was no change on the LC3B protein expression with confocal microscopy. The LC3B was detected only as a LC3B-I form in the pellet which, in accordance with the confocal findings, remained unaffected following irradiation (**Figure 1f**).

### IR suppresses the autophagy flux

Using double LC3A/LAMP2a and double LC3B/CathepsinD staining we confirmed a considerable degree of LC3A/LAMP2a (pc 0.73) and LC3B/CathepsinD (pc 0.71) co-localization in non-irradiated cells (**Fig. 1b** **1, 1c1**) implying a high rate of LC3A and LC3B autophagic flux. This was also supported by the limited presence of cytoplasmic p62 dots that indicates a high rate of p62 degradation after autophagolysosomal fusion (**Fig. 1d** **1**). Of interest, p62 was localized in the nuclei of cells; this implied a poorly understood, as yet, physiological role of the protein.

At 4 and 8 days following irradiation, reduction of LC3A/LAMP2a and of LC3B/Cathepsin D co-localization was recorded (pc <0.55), suggesting a suppression of the autophagolysosomal fusion (**Fig. 1b** **2,3 and 1c2,3**). LAMP2A and Cathepsin D expressions were increased in the cytoplasm, which indicated lysosome accumulation, presumably due to low lyosomal consumption rate (**Fig. 1e**). This was also confirmed with the western blot analysis (**Fig. 1f** **)**.

The above suggested suppression of autophagic flux was supported by the intensified cytoplasmic accumulation of the p62 protein, which was indicative of non-degraded autphagosome accumulation (**Fig. 1d** **2,3 and 1e**). This was confirmed by the pellet fraction of the western blot analysis (**Fig. 1f**). Nuclear p62 localization, evident in non-irradiated cells, significantly decreased after irradiation.

### Effect of IR on autophagy-regulating proteins

**Figure 2:**
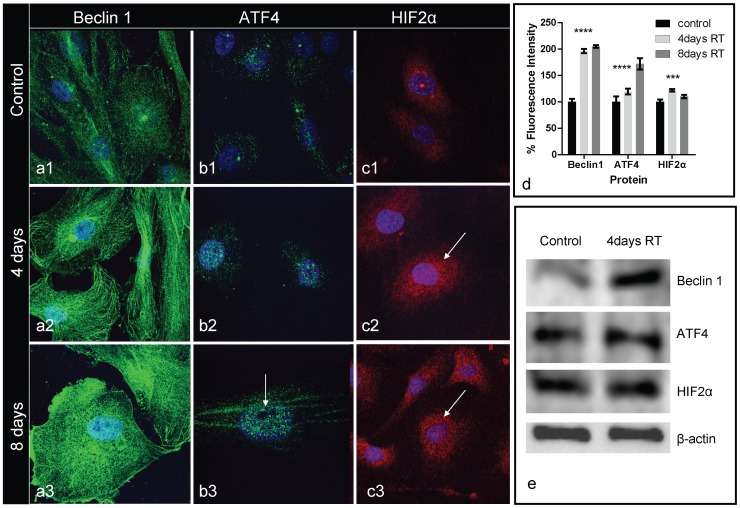
HUVEC confocal images of Beclin 1 (green) (Fig. 2a), ATF4 (green) (Fig. 2b) and HIF2α (red) (Fig. 2c). Intense accumulation of Beclin 1 was evident 4 and 8 days after irradiation (2a2, 2a3). Intense nuclear localization of ATF4 (arrows) at 4 and 8 post-irradiation days is shown in figure panel 2b, while HIF2α was also induced in the cytoplasm and nuclei (arrows) of HUVEC (figure panel 2c). Graphic presentation of fluorescent intensity of the above immunostaining is shown in Figure 1d. Figure 1e shows the western blot analysis in the pellet fraction of HUVEC.

Beclin 1, another autophagy-initiating factor, was highly up-regulated in the cytoplasm, and also in the nuclei of HUVEC, following IR exposure, assuming a ‘filament distribution’ (**Fig.** **2a** **1,2,3** and **2d**). Indeed, the western blot analysis showed accumulation of the Beclin 1 in the pellet fraction (**Fig. 2e**) and not in the supernatant.

Activating transcriptional factor 4 (ATF4) directly regulates the autophagy-initiating kinase ULK1(UNC51-like kinase1) under severe hypoxic and ER-stress [11]. ATF4 intensively translocated in the nuclei following irradiation (**Fig. 2b** **1,2,3**).

HIF2α (EPAS1; endothelial pass domain containing protein-1), a hypoxia inducible transcription factor essential for the development of the vascular system [12], was also up-regulated and accumulated in the cytoplasm and nuclei (**Fig, 2c, 2d** and **2e**). The HIF1α expression followed a similar, although less intense, pattern of response (data not shown).

### SMER28 enhances the autophagic flux

**Figure 3:**
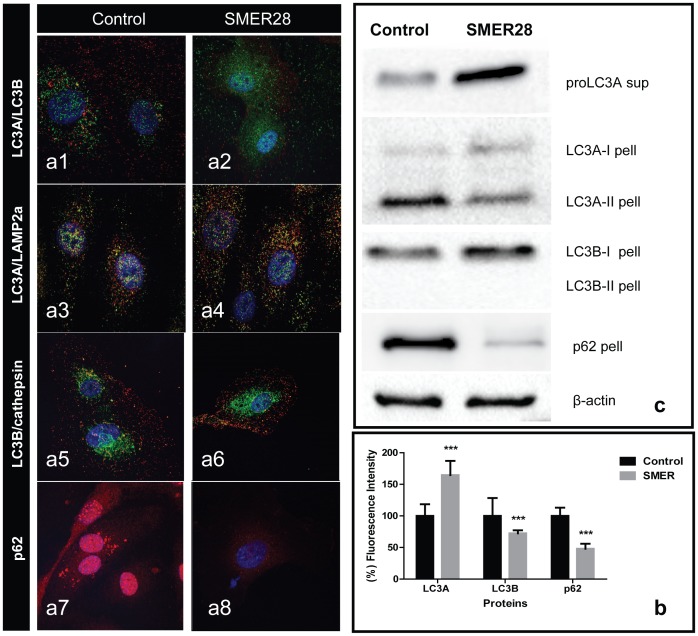
HUVEC confocal images LC3A(green)/LC3B(red) (Fig. 3a1,2), LC3A(green)/LAMP2a(red) (Fig. 3a3,4), LC3B(red)/CathepsinD(green) (Fig. 3a5,6) and p62 (red) (Fig. 3a7,8), before and after 2 days exposure to SMER28. Co-localization is shown as yellow structures. SMER28 induces expression of LC3A/LC3B and co-localization with lysosomal markers, while p62 cytoplasmic content is reduced. Graphic presentation of fluorescent intensity of the above immunostaining is shown in Fig. 3b. Figure 3c shows western blot images from the supernatant or pellet of HUVEC proteins.

The effect of SMER28 on autophagy flux was studied. SMER28 is a small-molecule enhancer of autophagy via an mTOR-independent and Atg5-dependent pathway. Following incubation with SMER28 (25–50 µΜ), LC3A autophagosomes were accumulated in the cytoplasm, while LC3B autophagosomes slightly reduced (**Fig. 3a** **1, a2** and **3b**). In the western blot analysis, cells exposed to SMER28 showed strong induction of the proLC3A form (**Fig. 3c**), which is suggestive of an acceleration of proLC3 production to form soluble LC3-I proteins.

Double LC3A/LAMP2a and LC3B/CathepsinD staining demonstrated an intensified co-localization after exposure to SMER28 (R>0.71), which is an indication of LC3A and LC3B autophagolysosomes and accelerated autophagic flux (**Fig. 3a** **3, a4** and **3a5, a6**). p62 was drastically reduced in the cytoplasm, which indicates degradation through the intensified autophagolysosomal fusion (**Fig. 3a** **7, a8** and **3b**). This was confirmed by the western blot analysis (**Fig. 3c**). The reduction of the LC3A-II membrane-bound protein was also evident in the western blot of the pellet fraction (**Fig. 3c**), confirming increased autophagic flux. However, we were not able to detect the LC3B-II 16 kDa form.

### SMER28 sustains the autophagic flux after irradiation

**Figure 4:**
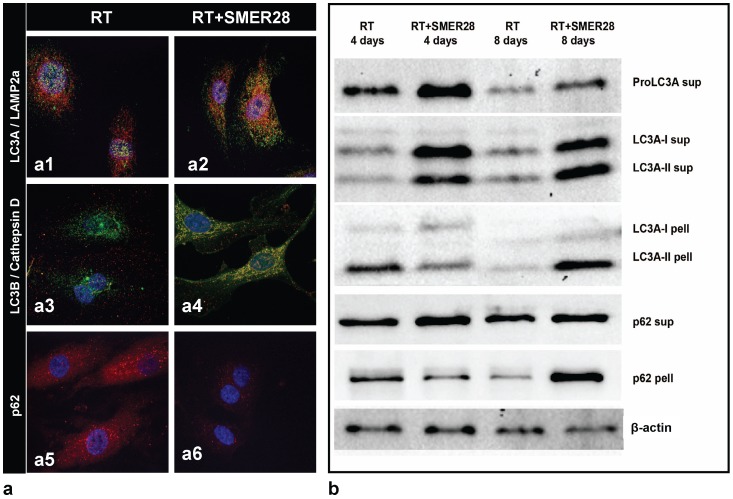
Confocal images of HUVEC cultured cells after exposure to 2 Gy of IR, with and without 2 day pre-incubation with SMER28 (Fig. 4a). LC3A(green)/LC3B(red) (Fig. 3a1,2), LC3A(green)/LAMP2a(red) (Fig. 3a3,4), LC3B(red)/CathepsinD(green) (Fig. 3a5,6) and p62(red) (Fig. 3a7,8). Co-localization is shown as yellow structures. Western blot images from supernatant and pellet protein fractions is shown in Fig. 4b.

Comparative assessment of double LC3A/LAMP2a, LC3B/CathepsinD and of p62 immunostaining, 4 and 8 days after irradiation, was performed with vs. without incubation with SMER28. This revealed that SMER28-treated cells sustained a good autophagic flux supported by the intense LC3A –LAMP2A (**Fig. 4a** **1, 3a2**) and LC3B-CathepsinD (**Fig. 4a** **3, 3a4**) co-localization and the reduction of cytoplasmic p62 (**Fig. 4a** **5, 4a6**). The Pearson coefficient for co-localization 4 days following irradiation was 0.63 and 0.45 without SMER28 for LC3A/LAMP2A and LC3B/Cathepsin D pairs, respectively, while this increased to 0.72 and 0.75, respectively, when cells were incubated with SMER28.

The western blot analysis (**Fig. 4b**) revealed that irradiated cells showed intense expression of proLC3A when pre-incubated with SMER28, suggesting a protective effect of the compound on LC3A production. At 4 post-irradiation days, the LC3A-I and II forms were over-expressed in SMER28 pretreated irradiated cells in the soluble fraction showing an intense release of the LC3A-II form from autophagosomes back to the cytoplasm, which is indicative of increased autophagic flux. This was also supported by the reduced LC3A-II and p62 expression in the pellet fraction. Also, at 8 days after irradiation, SMER28 pre-treated cells sustained an intense presence of LC3A-I and II forms in the supernatant, while LC3A-II and p62 intensively reappeared in the pellet fraction. This result presumably reflected an intensified formation of autophagosomes and rebound production of p62. This did not occur in cells receiving irradiation only, where LC3A and p62 proteins remained poorly expressed in both supernatant and pellet fractions.

### Response to IR

**Figure 5:**
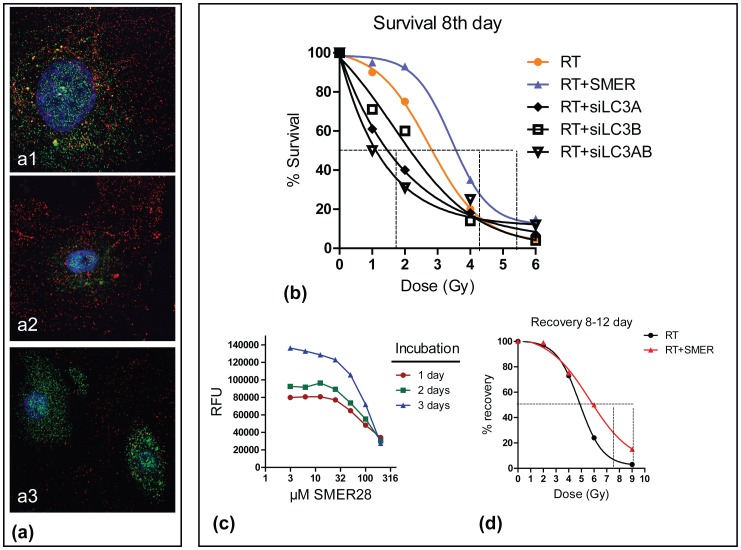
Confocal microscopy for LC3A/LC3B, specific suppression of LC3A (Fig. 5a2) and of LC3B (Fig. 5a3) after incubation with relevant LC3 siRNAs (Fig. 5a). Radiation dose and response curves confirm a shift to the right when a suppression of the LC3A/LC3B expression is achieved before irradiation, while the curve shifts to the right when cells are pre-incubated with non-toxic doses of the mTO- independent autophagy inducer SMER28 (Fig. 5b). Viability curves of HUVEC cells after exposure from 1–3 days at various SMER28 concentrations confirm nontoxic effect at 25 µM (Fig. 5c). Radiation dose and recovery curves comparing the cell population at the nadir (8^th^ day) vs. exponential growth onset (12^th^ day) show enhanced recovery potential when cells are pre-incubated with SMER28 (Fig. 5d).

Incubation with specific LC3 siRNAs was used to assess changes in radiation sensitivity. **Figures 5a** **1, a2, a3** show confocal images of LC3A and LC3B reduction as a result of appropriate siRNA treatment. Radiation dose/response analysis showed left displacements of the curves, especially in case of double silencing, with an estimated half-maximal inhibiting dose ID50 (50% survival) at 2.8 Gy for control cells vs. 1.1 Gy for double siLC3A, B treated cells; **Fig. 5b**.

Viability and titration experiments were performed with SMER28 for bewteen 1 and 3 days by daily monitoring. SMER28 concentrations ≤25 µΜ had no apparent effect on HUVEC viability, following 3-day exposure (**Fig. 5c**), while 50 µΜ SMER28 demonstrated a slight viability reduction of 20%. Control and cells treated with 25 µΜ SMER28 for 2 days were irradiated in 96-well plates with escalated radiation doses. The survival of cells was recorded (in terms of RFU) at 8 days (where a nadir of cell counts is noted) and at 12 days (where re-growth of cells is restored). At 8 days, the half-maximal inhibiting dose ID50 (50% survival) was estimated at 2.8 Gy for control cells and 3.5 Gy for SMER28 treated cells (**Fig. 5b**). At 12 days, the dose allowing 50% recovery of the cell population present at 8 days (RD50) was estimated at 5 Gy in control cells and 6 Gy in SMER28 pretreated cells (**Fig. 5d**).

### SMER does not protect against IR-induced γH2AX foci formation

**Figure 6:**
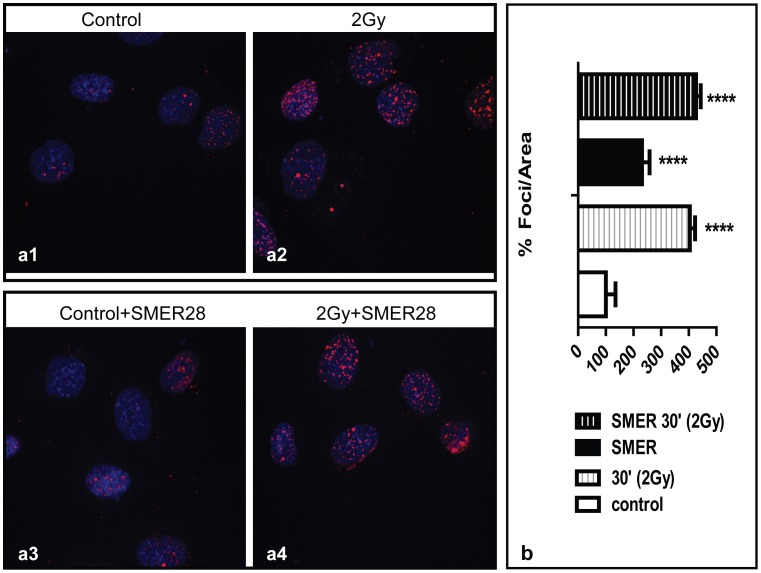
Confocal microscopy after γΗ2ΑX immunostaining, showing red foci in the nuclei of control (6a1,6a3) and irradiated cells (6a2,6a4), with (6a3,6a4) and without (6a1,6a2) pre-incubation with SMER28 (Fig. 6a). Computerized calculation of γH2AX foci in the nuclei of cells. Statistical significance refers to comparison with control cells (Fig. 6b).

In an attempt to identify whether SMER28 protects HUVEC against irradiation by reducing DNA damage, the formation of γΗ2ΑΧ nuclear foci at 30 min after irradiation was calculated using a computerized method. γΗ2ΑΧ foci increased at a similar rate whether or not the cells were pretreated with SMER28 (**Fig. 6**). Exposure to SMER28 slightly increased the formation of γΗ2ΑΧ foci in non-irradiated cells.

## Discussion

Autophagy ensures cell homeostasis by degrading damaged organelles and proteins and by simultaneously providing energy under stressful conditions. Although IR damages the DNA, cytoplasmic organelles are also damaged [13]. The effect of IR on the autophagy machinery has not been thoroughly examined. Most studies on cancer cells report how blockage or induction of autophagy affects their response to radiation [7], but its effect on autophagy remains elusive. Paglin et al. first identified the formation of acidic vacuoles in neoplastic epithelial cells exposed to IR [14]. Ito et al. interpreted the IR-induced accumulation of the LC3-II membrane form in glioma cells in western blots as induction of autophagy [15]. Liang et al. using Hela cells suggested that radiation induces autophagy, after having noticed an increase of LC3-II/LC3-I ratio [16]. For normal tissues, there are only sporadic data on how IR affects autophagy at therapeutic doses. Gorbunov et al. reported an autophagy increase in the crypt cells of the murine small intestine 7 days after the exposure to γ-radiation [17].

Nonetheless, the above evidence of the radiation induction of autophagy should be regarded with caution as accumulation of LC3 protein in cells can be indicative of either increased autophagic flux (enhanced autophagosome formation and processing) or blocked autophagy (accumulation of autophagosomes due to lack of autophagolysosomal processing) [18]. Fractionation of cell/tissue material in membrane and soluble fraction is not routinely performed in published studies, which results in complicated interpretation of western blots. Indeed, immunobloting has shown that the LC3A-I and LC3A-II protein levels increased significantly at 72 h and 7 days following irradiation in mouse lung tissues only in the cytosolic fraction, whilst these remained unchanged in the membrane fraction [19]. Similarly, p62 increased and the LC3A mRNA levels significantly declined. The above pattern of autophagic response was considered an evidence of autophagosomal down-regulation and dysfunction, but whether this was due to defective maturation or to aberrant degradation of the autophagosomes remained unclear.

Confocal microscopy is a potent tool to identify autophagic flux [18]. Co-localization of autophagosomal and lysosomal proteins is useful to identify the fusion and, presumably, the functionality of the process. Using this method accumulation of autophagosomes vs. accumulation of autophagolysosomes can be readily identified. In the current study, using confocal microscopy with double immunostaining for autophagosomal and lysosomal proteins, we identified a suppression of the autophagic flux 4 and 8 days following irradiation. LC3A and LC3B positive autophagosomes were accumulated without any fusion with lysosomes. LAMP2a and CathepsinD were also increased, which suggests reduced lysosomal consumption. Moreover, p62 cytoplasmic concentration was increased, which suggests deceleration of recycling of the major carrier of material to autophagosomes.

Radiation also exhibited an intense effect on the expression of certain transcription factors and proteins involved in autophagy initiation and regulation. ATF4, an important transcription factor regulating the expression of the ULK1 [11], an autophagy initiating protein, was intensively translocated into the nuclei of cells. HIF2α endothelial cell specific hypoxia inducible factor, and also HIF1α, were also up-regulated by radiation and translocated into the nuclei. The link between autophagy and HIFs is complex. Bohensky et al. suggested that HIF2α strongly suppresses autophagy in certain cells like chondrocytes [20]. HIF1α on the other hand is an important positive regulator of autophagy [21]. In the current study, the autophagy-initiating factor Beclin 1 was clearly accumulated in the cells following irradiation. In any case, this orchestrated up-regulation of transcription factors related to autophagy are in the context of a survival/protection cell reaction which, although may contribute to endothelial cell protection, fails to overcome the blockage of autophagic flux induced by radiation. The pathway leading to autophagolysosomal flux repression after irradiation remains elusive, but certainly the current data clarify certain aspects by excluding an eventual repressive effect of radiation on the transcriptional factor and autophagy-initiating protein expression.

In order to investigate the eventual effect of a forced induction of autophagy flux on radiation cytotoxicity in HUVEC, we used the SMER28, a small-molecule mTOR independent agent reported to induce functional autophagy via the ATG5 pathway in neuronal cell cultures [8], and avoided an mTOR dependent enhancer of autophagy. mTOR inhibition may have multiple effects on biological pathways sensitizing cells to radiation [22], which had to be avoided. SMER28 had a robust affect on endothelial cell autophagic response at non-toxic dose levels. Co-localization of autophagy and lysosomal markers was highly induced by SMER28, and p62 was drastically reduced in the cytoplasm. This result confirmed acceleration of the autophagic flux. When SMER28 preceded irradiation, the autophagic flux supression was prevented, and was accompanied by increased resistance of endothelial cells to radiation. On the contrary, silencing the LC3A and LC3B gene expressions with siRNAs resulted in enhancement of the toxic radiation effect. As irradiation at the current administered dose levels suppresses, but not entirely abrogates, the autophagic flux, concurrent compromisation of the autophagy function by reducing LC3 protein content seems to enhance radiation cell killing. Whether high radiation doses can totally block the autophagic flux, and how this may be related to tissue-specific responses to different radiotherapy fractionation, are issues that demand further investigation. An eventual effect of SMER28 protecting against DNA damage was excluded by experiments showing no protection against γH2AX nuclear foci formation.

The present study provides for the first time strong evidence that ionizing radiation at therapeutic dose levels results in prolonged repression of the autophagic flux in endothelial cells. This can be prevented by the exposure of cells to mTOR-independent enhancers of autophagy like SMER28 at nontoxic concentrations. Protection of the autophagic flux results in endothelial cell protection against radiation damage. These data provide a strong rationale for the development of cytoprotection policies that would have a great impact in the quality of life of patients undergoing curative radiotherapy, or even in the curability of cancer since such policies would allow the safe administration of higher radiotherapy doses.
